# Management of Mild to Moderate Pain from Triage to Discharge in the Emergency Department: A Multidisciplinary Delphi Consensus from the Italian Society of Emergency Medicine (SIMEU)

**DOI:** 10.3390/jcm15093230

**Published:** 2026-04-23

**Authors:** Alessandro Riccardi, Fabio De Iaco, Elena Del Giudice, Mario Guarino, Niccolò Parri, Federico Pea, Antonella Cocorocchio, Sossio Serra

**Affiliations:** 1 SAU Working Group, Italian Society of Emergency Medicine (SIMEU), Via Valprato 68, 10155 Turin, Italy; 2 Unit of Emergency Medicine, Ospedale Santa Corona, Via XXV Aprile, 38, 17029 Pietra Ligure, Italy; 3 Emergency Medicine and Urgent Care Unit 1, Maria Vittoria Hospital, ASL Città di Torino, Via Cibrario 72, 10144 Turin, Italy; 4 Geriatrics Unit, San Giovanni Addolorata Hospital, Via dell’Amba Aradam 8, 00184 Rome, Italy; 5 Complex Unit of Emergency Medicine and Urgent Care (UOC MEU), CTO Hospital, AORN dei Colli, Viale Colli Aminei, 21, 80131 Naples, Italy; 6 Department of Emergency Medicine and Trauma Center, Meyer Children’s Hospital IRCCS, Viale Pieraccini 24, 50139 Florence, Italy; 7 Department of Medical and Surgical Sciences, Alma Mater Studiorum, University of Bologna, Via Irnerio 49, 40126 Bologna, Italy; 8 Clinical Pharmacology Unit, Department for Integrated Infectious Risk Management, IRCCS Azienda Ospedaliero-Universitaria di Bologna, Via Massarenti 9, 40138 Bologna, Italy; 9 Emergency Department Nursing Staff, Azienda Ospedaliera San Giovanni Addolorata, Via dell’Amba Aradam 8, 00184 Rome, Italy; 10 Nursing Area, Italian Society of Emergency Medicine (SIMEU), Via Valprato 68, 10155 Turin, Italy; 11 Complex Unit of Emergency Department and Emergency Medicine (UOC), Ramazzini Hospital, Via Molinari 2, 41012 Carpi, Italy

**Keywords:** emergency medicine, mild-to-moderate pain, pain management, multimodal analgesia, paracetamol, ibuprofen, Delphi consensus

## Abstract

**Background:** Mild and moderate pain represent a large proportion of emergency department (ED) presentations but are frequently underestimated and inconsistently managed, particularly in vulnerable populations such as children and older adults. Standardised and evidence-based approaches are needed to ensure timely, safe, and effective pain control across the entire emergency care pathway. **Methods:** A national multidisciplinary Delphi consensus was conducted under the auspices of the Italian Society of Emergency Medicine (SIMEU). A Scientific Steering Committee performed a systematic literature review and developed 26 statements comprising 92 items across four thematic areas: analgesia at triage, risk factors and analgesia at discharge, analgesia in children, and analgesia in elderly patients. Thirty-three experts from across Italy participated in three Delphi rounds, rating each item using a five-point Likert scale. Consensus was defined as ≥66% agreement (scores 4–5). **Results:** Consensus was achieved for 78 out of 92 items. Key recommendations include early pain assessment at triage using validated scales, paracetamol as first-line therapy for mild and moderate pain across all age groups, and the use of multimodal analgesia for moderate pain. Fixed-dose combinations of paracetamol and ibuprofen were strongly endorsed for their efficacy, safety, and opioid-sparing effect in adults, children, and elderly patients. Clear guidance was also provided for analgesic selection at discharge, duration of therapy, patient education, and management of special populations. **Conclusions:** This multidisciplinary Delphi consensus provides practical, evidence-based recommendations to harmonize the management of mild and moderate pain in ED. Implementation of these recommendations may improve pain control, patient safety, and quality of care in non-urgent emergency settings.

## 1. Introduction

Mild and moderate pain is a common yet often underestimated challenge in emergency medicine. According to European Society for Emergency Medicine (EUSEM) recommendations, mild and moderate pain are defined as Numerical Rating Scale (NRS) scores of 1–3 and 4–6, respectively. Mild and moderate pain occurs in conditions such as musculoskeletal trauma, headache, abdominal pain, and postoperative discomfort, but is frequently undertreated or inadequately assessed. This may result in prolonged distress, delayed recovery, and reduced patient satisfaction [1,2]. Traditionally, Emergency Departments (EDs) prioritise severe pain (NRS ≥ 7), leaving moderate pain inconsistently managed. Recent Italian guidelines stress early assessment and standardised analgesic protocols across triage levels to ensure equitable and effective pain care [3].

Italy’s ageing population, projected to exceed 30% of individuals aged over 65 years by 2051, is reshaping the pain management strategies [4,5]. Older adults frequently present frailty, multimorbidity, and polypharmacy, which complicate treatment. Age-related pharmacokinetic and pharmacodynamic changes heighten susceptibility to adverse drug reactions, particularly those associated with Non-Steroidal Anti-Inflammatory Drugs (NSAIDs) and opioids [6,7]. NSAIDs may cause gastrointestinal bleeding, renal impairment, and cardiovascular toxicity [8,9]. Opioids also require cautious dosing, as they are associated with sedation, confusion, falls, and constipation [10,11]. Consequently, paracetamol remains the preferred first-line option for mild pain because of its favourable efficacy and safety [12,13]. The use of NSAIDs should be carefully assessed and limited to situations in which they are strictly necessary. They should be used at the lowest effective dose for the shortest duration and avoided in high-risk patients [7,9]. The fixed-dose combination (FDC) of paracetamol and ibuprofen is the preferred choice for moderate pain in older adults because of the synergistic action of the two active principles, as discussed below.

At the opposite end of the age spectrum, paediatric pain management presents specific challenges. Pain may be underestimated due to difficulty in assessing non-verbal or cognitively impaired children, limited use of validated scales, and misconceptions about pain tolerance [14,15,16]. Inadequate analgesia can increase pain sensitivity, anxiety, and non-compliance during future procedures [16]. Developmental differences in absorption and metabolism further affect drug response and toxicity [17]. Paracetamol remains the drug of choice for mild pain, while NSAIDs must be used cautiously in all patients with special attention to dehydrated or renally compromised children [18].

Across all ages, ensuring appropriate and safe analgesic treatment is critical. A major advancement is multimodal analgesia, which combines drugs acting on different pain pathways to enhance efficacy and reduce adverse effects [2,19]. Among these strategies, the FDC of paracetamol and ibuprofen is a recommended option for moderate pain. The FDC leverages the synergistic interaction between paracetamol’s central action and ibuprofen’s predominantly peripheral effect. The FDC of paracetamol and ibuprofen provides additive analgesic effects, allows a reduction in the required NSAID dose, and offers faster onset and greater efficacy than either drug alone [20,21,22,23]. Pharmacokinetic data show improved absorption and longer-lasting analgesia [24]. In older adults, FDCs of paracetamol and ibuprofen optimise pain relief with good tolerability [7,25]. In children these combinations enhance pain control and reduce dosing errors [18,26]. Despite strong evidence, multimodal strategies remain underused in EDs, highlighting the need for education and consistent implementation [27,28,29].

To address these gaps, the Italian Society of Emergency Medicine (SIMEU) launched a national multidisciplinary Delphi consensus to promote appropriate, safe, and uniform management of mild and moderate pain in non-urgent care. This initiative aimed to define the extent of the problem, identify key challenges across the care pathway, promote rational and safe analgesic use through expert discussion, and develop a shared, evidence-based consensus document for emergency professionals.

## 2. Methods

### 2.1. Preparatory Phase and Questionnaire Set Up

The Scientific Steering Committee is composed of five SIMEU members (four emergency physicians and one nurse), one geriatrician, one paediatrician and one pharmacologist. The Scientific Steering Committee identified the scientific areas to be explored in the project.

A comprehensive literature review was conducted on PubMed, ClinicalTrials.gov, Cochrane Database Systematic Reviews to retrieve full-text articles published on humans in the last ten years [metanalysis of randomised controlled trials (RCTs), papers on RCTs, literature reviews, scientific guidelines]. The literature search strategy was based on PICO method and is displayed in Supplementary Methods.

The retrieved papers were assessed for their level of evidence by the Scientific Steering Committee; for each topic, the Committee selected the papers with the highest level of evidence based on judgment of the experts and the OCEBM Levels of Evidence—Centre for Evidence-Based Medicine (CEBM), University of Oxford.

The literature search was complemented by Italian consensus documents addressing organisational aspects of emergency care.

In line with the national scope of the project, the statements were developed considering the organisation, resources, and clinical practices of Italian EDs. Based on the results of the literature review and appraisal of the retrieved papers, the Scientific Steering Committee organised the content into the following four thematic areas, comprising a total of 26 statements and 92 items.

Thematic area 1—Analgesia at triage (10 statements, 38 items);

Thematic area 2—Risk factors and analgesia at discharge (7 statements, 24 items);

Thematic area 3—Analgesia in children with mild-to-moderate pain (5 statements, 14 items);

Thematic area 4—Analgesia in elderly patients with mild-to-moderate pain (4 statements, 16 items).

Supplementary Table S1 displays the 92 items validated by the Scientific Steering Committee.

### 2.2. Delphi Rounds and Voting Procedure

The Delphi voting was conducted during a meeting held in May 2025 in Bari. Thirty-three experts across Italy (see Supplementary Table S2) voted in the Delphi rounds, while the members of the Scientific Steering Committee attended the meetings without casting their vote. The panellists participated in three voting rounds rating their level of agreement on each item by a five-point Likert scale (1 = strongly disagree; 2 = disagree; 3 = uncertain; 4 = mostly agree; 5 = strongly agree).

### 2.3. Definition of Consensus Conditions

Responses collected through the Likert scale were divided into three scoring groups of increasing agreement (Table 1): 1–2 strongly disagree/disagree, 3 uncertain, and 4–5 mostly agree/strongly agree.

The agreement condition (positive consensus) was defined when at least 66% of panellists’ responses clustered within the 4–5 scoring group.

A disagreement condition (negative consensus) was defined when at least 66% of panellists’ responses clustered within the 1–2 scoring group.

Other distributions of votes are defined as “consensus not reached”.

At the end of the first round, items that reached positive consensus were included in the final recommendations. Those that did not reach consensus were revised based on the panel’s comments and resubmitted for the following voting rounds. All responses were collected anonymously and aggregated to calculate the percentage of agreement and disagreement for each item.

## 3. Results

At the end of the Delphi process, agreement was achieved for 78 of the 92 items, which were included in the final set of recommendations (Supplementary Table S3). Of the remaining 14 items, 12 did not reach agreement and two were excluded. Table 2 provides an overview of the number of items achieving agreement, in disagreement or excluded across the four thematic areas.

The results divided by thematic area are reported below.

**Thematic area 1—Analgesia at triage:** Thirty-three out of 38 items reached the agreement; of the remaining five items, four resulted in disagreement (items 5.3, 5.4, 5.5, 7.2) and one was deleted (item 9.4).

**Thematic area 2—Risk factors and analgesia at discharge:** Agreement was reached for 19 of 24 items; of the remaining five items, four were in disagreement (negative consensus) (items 12.3, 13.1, 14.1 and 17.4) and one (item 14.3) was eliminated.

**Thematic area 3—Analgesia in children with mild-to-moderate pain:** Agreement was reached for all the 14 items.

**Thematic area 4—Analgesia in elderly patients with mild-to-moderate pain**: Of the 16 items, 12 reached the agreement, while four items remained in disagreement.

Items that did not reach consensus or were excluded (Table 3) mainly concerned pharmacological strategies characterized by heterogeneous risk-benefit perceptions, variability in clinical practice, or limited agreement on their appropriateness within the scope of the consensus. The reasons for disagreement or exclusion are reported in Supplementary Tables S4 and S5. The set of 78 final items is presented (Table 4, Table 5, Table 6 and Table 7) and discussed by thematic area in the Section 4.

## 4. Discussion

### 4.1. Thematic Area 1—Analgesia at Triage

Standardised criteria, such as the reason for admission and patient-specific characteristics, are essential to guide analgesic administration and ensure equitable management [1,3,30].

Paracetamol is recommended as the first-line analgesic across all groups of patients due to its broad therapeutic index and well-documented hepatic and renal tolerability [13,31,32]. Its favourable risk-benefit profile is an advantage in all patients, including the settings where safety considerations are paramount. This is reflected in statement 3: “Paracetamol should be considered the first-line choice for the pharmacological management of mild and moderate pain at triage in vulnerable populations, due to its favourable safety profile.”

In contrast, NSAIDs, while effective, require careful consideration of contraindications and comorbidities, particularly gastrointestinal, renal, and cardiovascular conditions [33,34].

Available evidence supports the role of multimodal analgesia, particularly through FDCs of paracetamol and ibuprofen. These combinations enhance efficacy and prolong the duration of analgesia while minimizing the need for higher individual doses [2,21,22]. In clinical practice, these FDCs are available in several formulations; the FDC containing paracetamol 1000 mg and ibuprofen 300 mg (up to three times daily) is widely used in Italy and recommended by the Italian Society of Orthopaedics and Traumatology (SIOT) for the pain management [35].

Sublingual sufentanil should be maintained as an optional intervention for moderate pain and adaptable to each ED’s resources and patient feature. Its advantages include rapid onset, high potency and the possibility of administration by nurses. However, its use is limited by reduced experience in EDs and cost [3,36].

Although patient reassessment is an integral part of triage protocols, clinical practices differ in timing, frequency, and scope. This variability is particularly evident in vital sign monitoring and in the management of partial pain relief. The panel stressed the need for standardised guidance on patient monitoring to ensure consistent, safe, and effective analgesic management across EDs. In this regard, the Italian SIAARTI/SIMEU intersocietal guidelines [3] emphasise structured reassessment and documentation as essential components of quality care, in line with international literature [37,38,39]. Continuous staff training and institutional protocols are also deemed essential to overcome barriers, such as overcrowding and understaffing [37,38].

### 4.2. Thematic Area 2—Risk Factors and Analgesia at Discharge

Discharge analgesia should be considered as an extension of emergency care, demanding both pharmacological prudence and patient empowerment. The treatment at discharge should consider comorbidities and other patient characteristics. As stated in item 11.1, “Paracetamol should be the first-line drug at discharge for adults with mild-to-moderate pain due to its favourable safety and efficacy profile”, also in patients with comorbid hepatic, renal, cardiovascular, or gastrointestinal disease [12,13,40].

The NSAIDs, useful in presence of inflammatory component, should be used at the lowest effective dose for the shortest possible duration, particularly in elderly patients or those with organ dysfunction [33,34]. As outlined in item 13.4 “The fixed-dose combination of paracetamol and ibuprofen represents a valid therapeutic option for moderate musculoskeletal or traumatic pain”. Combining paracetamol with an NSAID offers additive efficacy and reduced NSAID exposure, minimising the adverse events related to NSAID [22]. Among the options available at discharge, FDCs such as paracetamol 1000 mg plus ibuprofen 300 mg (up to three times daily) provide effective analgesic therapy because of the synergistic action of the active principles [35]. Weak opioids, including codeine and oxycodone in FDCs, should be considered as second-line agents for patients intolerant or unresponsive to first-line drugs [41,42,43]. However, they must be administered for short-term (3–5 days) and subjected to follow-up to avoid dependency and adverse effects. Opioid overprescription at discharge contributes to avoidable readmissions and misuse, reinforcing the need for strict monitoring [3].

Conventional analgesics are usually ineffective for neuropathic pain; therefore, medications acting on neuropathic mechanisms, such as gabapentinoids, antidepressants, or tramadol-like agents, should be preferred [44,45].

Patient and caregiver education are a critical determinant of safety. Providing clear, written instructions on dosing, duration, and warning signs, together with information on non-pharmacological strategies and when to seek reassessment, enhances adherence and prevents medication errors [46].

### 4.3. Thematic Area 3—Analgesia in Children with Mild-to-Moderate Pain

Paracetamol is confirmed as the first-line therapy for mild-to-moderate pain across all age groups (0–18 years), because of its predictable pharmacokinetics, wide safety margins, and proven efficacy [15,47]. The oral or orodispersible routes are preferable, balancing efficacy, practicality, and acceptance [3].

Ibuprofen may also be used from three months of age in moderate pain or when an inflammatory component is suspected [15,48]. However, its use requires a careful clinical assessment. In paediatric patients, paracetamol remains the preferred option given its more favourable safety profile.

As stated in item 19.1, “When greater pain control is needed, a fixed-dose combination of paracetamol and ibuprofen should be preferred.” This combination is a valuable therapeutic option, as it allows effective analgesic control of moderate pain while limiting NSAIDs exposure and dosing errors. This is particularly advantageous in emergency contexts [18,26].

Standardised dosing regimens are recommended. Paracetamol is administered at 15 mg/kg per dose, with scheduled rather than “as needed” administration, to maintain stable plasma levels; ibuprofen is administered at 5–10 mg/kg every eight hours. FDC of paracetamol and ibuprofen is administered according to a dosage table that allows a dosage of 12 mg/kg of paracetamol and 3.6 mg/kg of ibuprofen.

Opioids, such as tramadol, may be reserved for cases unresponsive to first-line agents. In these cases, rigorous clinical monitoring is required to prevent respiratory or neurological adverse events [15].

Although the oral or orodispersible routes are preferable, the rectal and intravenous administrations route remain relevant in paediatric practice, particularly when oral administration is not feasible. This reflects the broader challenge of route selection in children, whereas intravenous administration can be adopted more readily for adults in the emergency setting [49].

Finally, the communication with caregivers is crucial for therapy adherence and emotional support. Shared decision-making reduces anxiety, improves cooperation, and increases treatment effectiveness, especially in non-verbal or anxious children [16].

### 4.4. Thematic Area 4—Analgesia in Elderly Patients with Mild-to-Moderate Pain

Pain management in elderly patients presents specific challenges, given the prevalence of frailty, polypharmacy, and physiological changes that alter drug metabolism and response [6,10]. The patient assessment should be individualised and based on validated tools such as the Numeric Rating Scale (NRS), Verbal Rating Scale (VRS), and PAINAD for patients with cognitive impairment [25].

Paracetamol remains the first-line therapy for mild-to-moderate pain in elderly patients due to its favourable safety profile, low incidence of gastrointestinal or renal adverse events, and reliable efficacy across comorbid and frail patients [15,50]. NSAIDs may be used cautiously in selected patients because of the risk of gastrointestinal ulceration, renal dysfunction, and heart failure exacerbation [33]. Their use should be limited to short treatments and gastroprotection should be considered when indicated [7].

Multimodal analgesia is a mainstay of the treatment for elderly patients. FDCs of paracetamol and ibuprofen provide additive efficacy and a longer duration of pain relief, with pharmacokinetic advantages such as longer absorption and prolonged effect [22]. FDC of paracetamol 1000 mg and ibuprofen 300 mg is the treatment of choice for moderate pain. The synergistic action of the two drugs enables effective analgesia while reducing the required NSAID dosage. For cases requiring enhanced analgesia, combinations of paracetamol with low-dose oxycodone may offer effective control while maintaining acceptable safety [42]. Conversely, the panellists recommended against the combinations involving tramadol or codeine in frail or cognitively impaired patients. These combinations are associated with sedative and constipating effects, which increase fall risk and cognitive decline [3,51].

Finally, the panellists stressed the need for patient and caregiver education, simplified dosing regimens, and active adverse event monitoring. Involving caregivers improves adherence, minimizes medication errors, and ensures early recognition of adverse reactions [25].

## 5. Conclusions

This national Delphi consensus developed by SIMEU addresses a highly relevant yet often under recognised aspect of emergency care: the standardised management of mild and moderate pain. Panellists agreed on early pain recognition, rational analgesic selection, and the central role of paracetamol as first-line therapy across all patient groups. The consensus also endorsed multimodal analgesia for the management of moderate pain, including the use of FDCs of paracetamol 1000 mg and ibuprofen 300 mg, a formulation commonly adopted in Italian emergency practice.

Reflecting the organisation, resources, and clinical practices of Italian EDs, the recommendations are designed to be directly applicable in real-world clinical settings. This consensus thus provides practical, evidence-based guidance to harmonise mild and moderate pain management in EDs in Italy. Future efforts should focus on implementation, staff training, and real-world evaluation of outcomes.

## Figures and Tables

**Table 1:** Categorization of Likert scores by scoring groups.

Score Group	Strongly Disagree—Disagree	Uncertain	Mostly Agree—Strongly Agree
**Relevance**	Very low or low	Uncertain	High or very high
**Likert scale**	1–2	3	4–5

**Table 2:** Distribution of Delphi consensus outcomes across thematic areas.

Thematic Area	No. Initial Items	No. Items in Agreement	No. Items in Disagreement	No. Excluded Items
**1—Analgesia at triage**	38	33/38	4/38	1/38
**2—Risk factors and analgesia at discharge**	24	19/24	4/24	1/24
**3—Analgesia in children with mild-to-moderate pain**	14	14/14	0	0
**4—Analgesia in elderly patients with mild-to-moderate pain**	16	12/16	4/16	0

**Table 3:** List of items in disagreement or excluded from the final set of recommendations.

THEMATIC AREA 1—ANALGESIA AT TRIAGE	
**Drugs appropriate for mild pain at triage** Statement #5—“I believe that the following drugs are appropriate, in terms of risk/benefit ratio, for analgesia at triage in patients with mild pain.”	
5.3 Fixed-dose combination of paracetamol + ibuprofen	DISAGREEMENT
5.4 Fixed-dose combination of paracetamol + codeine	DISAGREEMENT
5.5 Sublingual sufentanil	DISAGREEMENT
**Route of administration** Statement #7—“I believe that, based on currently available formulations:”	
7.2 The sublingual route should be considered an alternative for managing mild pain at triage, particularly for its rapid onset of action.	DISAGREEMENT
**Organization and training** Statement #9—“I believe that:”	
9.4 ED overcrowding may represent a barrier to the implementation of the triage pain-management protocol.	EXCLUDED
**THEMATIC AREA 2—RISK FACTORS OF ANALGESIC THERAPY**	
**Drug-selection criteria at discharge** Statement #12—“I believe that:”	
12.3 Analgesic therapy at discharge should not be personalised according to clinical profile.	DISAGREEMENT
**NSAID use** Statement #13—“I believe that:”	
13.1 NSAIDs should be preferred for moderate pain of musculoskeletal or post-traumatic origin.	DISAGREEMENT
Opioid therapy at discharge Statement #14—“I believe that:”	
14.1 Immediate-release oral oxycodone should be preferred at discharge for moderate pain, thanks to its rapid analgesic efficacy and good bioavailability.	DISAGREEMENT
14.3 The paracetamol/codeine combination is indicated for moderate pain when NSAIDs are contraindicated.	EXCLUDED
**Patient and caregiver education** Statement #17—“I believe that:”	
17.4 A standardised protocol for prescribing analgesic therapy at discharge is preferable.	DISAGREEMENT
**THEMATIC AREA 4—ANALGESIA IN ELDERLY PATIENTS WITH MILD TO MODERATE PAIN**	
**Pharmacological management in the elderly** Statement #24—“I believe that:”	
24.3 If pain is not controlled with paracetamol, adding tramadol is an effective and safe option.	DISAGREEMENT
**Fixed-dose combinations in the elderly** Statement #26—“I believe that:”	
26.4 In elderly patients, fixed-dose combination paracetamol/tramadol administered orally is a valid treatment option for the control of moderate pain.	DISAGREEMENT
26.5 In elderly patients, fixed-dose combination oral NSAIDs/codeine is a valid treatment option for the management of moderate pain.	DISAGREEMENT
26.6 In elderly patients, fixed-dose combination oral NSAID/tramadol is a valid treatment option for the management of moderate pain.	DISAGREEMENT

For ease of reading, thematic areas and main topics headings are reported in bold type.

**Table 4:** Final items—Thematic area 1.

THEMATIC AREA 1—ANALGESIA AT TRIAGE		
**Criteria for administering analgesics at triage** Statement #1—“The administration of analgesics at triage for patients with mild-to-moderate pain should be based on standardised criteria such as:”		
1.1 Reason for ED admission	100.00%	AGREEMENT
1.2 Patient-specific characteristics	100.00%	AGREEMENT
**Variations for vulnerable populations** Statement #2—“The management of analgesic therapy at triage should include specific variations for certain vulnerable populations:”		
2.1 Age (children and elderly)	96.97%	AGREEMENT
2.2 Pregnancy and breastfeeding	96.97%	
2.3 Specific pathologies and rare diseases	75.76%	
**Paracetamol as first-line choice** Statement #3—“Paracetamol should be considered the first-line choice for the pharmacological management of mild and moderate pain at triage in vulnerable populations, due to its favourable safety profile.”		
3.1 Age (children and elderly)	96.97%	AGREEMENT
3.2 Pregnancy and breastfeeding	100.00%	
3.3 Specific pathologies and rare diseases	78.79%	
**Contraindications to be assessed** Statement #4—“The definition of a triage analgesia protocol should include the assessment of certain contraindications, such as:”		
4.1 Hypersensitivity or allergy to the analgesic specified in the protocol.	100.00%	AGREEMENT
4.2 Presence of bleeding disorders or use of anticoagulants.	100.00%	
4.3 Concomitant use of drugs with potential interactions.	93.94%	
4.4 Difficulty in collecting the patient’s medical history.	100.00%	
**Drugs appropriate for mild pain at triage** Statement #5—“I believe that the following drugs are appropriate, in terms of risk/benefit ratio, for analgesia at triage in patients with mild pain.”		
5.1 Paracetamol	100.00%	AGREEMENT
5.2 Ibuprofen	90.91%	
**Drugs appropriate for moderate pain at triage** Statement #6—“I believe that the following drugs are appropriate, in terms of risk/benefit ratio, for analgesia at triage in patients with moderate pain.”		
6.1 Paracetamol	100.00%	AGREEMENT
6.2 Ibuprofen	100.00%	
6.3 Fixed-dose combination of paracetamol + ibuprofen	100.00%	
6.4 Fixed-dose combination of paracetamol + codeine	81.82%	
6.5 Sublingual sufentanil	81.82%	
**Route of administration** Statement #7—“I believe that, based on currently available formulations:”		
7.1 The oral route should be considered the most advantageous for managing mild-to-moderate pain at triage due to its simplicity, safety and good efficacy.	100.00%	AGREEMENT
7.3 The sublingual route should be considered an alternative for managing moderate pain at triage, particularly for its rapid onset of action.	93.94%	
7.4 The IV route should be considered appropriate at triage for patients with mild-to-moderate pain who cannot take oral or sublingual drugs.	100.00%	
7.5 Alternative routes of administration should be considered valid options in selected cases within the triage management of mild-to-moderate pain.	96.97%	
**Monitoring of analgesic therapy at triage** Statement #8—“I believe that:”		
8.1 The effectiveness of analgesic therapy at triage must be regularly monitored using validated pain assessment scales.	100.00%	AGREEMENT
**8.2** Monitoring should include patient observation and re-assessment.	100.00%	
**8.3** Monitoring should also include early identification of possible adverse effects of the administered therapy.	100.00%	
8.4 In case of partial or insufficient response to analgesic therapy at triage, pain should be re-evaluated and alternative strategies considered.	81.82%	
**Organization and training** Statement #9—“I believe that:”		
9.1 Specific training of healthcare staff is a key element to be included in the organization of a triage analgesia protocol.	100.00%	AGREEMENT
9.2 A triage analgesia protocol should define clear treatment pathways and ensure the immediate availability of necessary analgesic drugs.	100.00%	
9.3 Non-pharmacological approaches (e.g., immobilization, ice application) should be included in the organization of a triage analgesia protocol.	100.00%	
**Ethical and legal aspects** **Statement #10**—“*I believe that*:”		
10.1 Adequate pain management at triage is a patient’s right and a clinical duty.	100.00%	AGREEMENT
10.2 Analgesia at triage should be initiated on the basis of the patient’s implicit consent after adequate information.	100.00%	
10.3 The assessment, treatment and outcome of pain management must be documented.	100.00%	

For ease of reading, thematic areas and main topics headings are reported in bold type.

**Table 5:** Final items—Thematic area 2.

THEMATIC AREA 2—RISK FACTORS OF ANALGESIC THERAPY		
**Analgesia at discharge (adult patients)** Statement #11—“I believe that:”		
11.1 Paracetamol should be the first-line drug at discharge for adults with mild-to-moderate pain due to its favourable safety and efficacy profile.	100.00%	AGREEMENT
11.2 For moderate pain with an inflammatory component, NSAIDs should be prescribed with caution in elderly patients or those at gastrointestinal, renal, or cardiovascular risk.	100.00%	
11.3 For moderate pain in adults, paracetamol combined with an NSAID may be a valid option at discharge.	96.97%	
11.4 Weak opioids should be considered for moderate pain at discharge, reserved for patients with contraindications to NSAIDs and/or paracetamol.	96.97%	
**Drug-selection criteria at discharge** Statement #12—“I believe that:”		
12.1 Analgesic therapy at discharge should be guided by the assessment of individual risk factors.	100.00%	AGREEMENT
12.2 Concomitant therapies with potential drug interactions should guide the choice of the safest analgesic.	100.00%	
**NSAID use** Statement #13— “I believe that:”		
13.2 NSAIDs should be preferred for moderate pain due to renal or biliary colic.	100.00%	AGREEMENT
13.3 NSAIDs should be preferred for moderate pain from acute headaches (e.g., tension-type headache or migraine).	100.00%	
13.4 The fixed-dose combination of paracetamol and ibuprofen represents a valid therapeutic option for moderate musculoskeletal or traumatic pain.	100.00%	
**Opioid therapy at discharge** Statement #14—“I believe that:”		
14.2 The oxycodone/paracetamol combination is indicated for moderate pain resistant to paracetamol/ibuprofen.	87.88%	AGREEMENT
**Duration of therapy at discharge** Statement #15—“I believe that:”		
15.1 Analgesic therapy for moderate pain at discharge should generally be prescribed for no more than 3–5 days, unless clinically indicated otherwise.	100.00%	AGREEMENT
15.2 A longer duration should be considered for specific conditions such as neuropathic pain.	100.00%	
15.3 Patients should receive instructions for short-term clinical follow-up by primary care.	100.00%	
**Neuropathic pain** Statement #16 — “I believe that:”		
16.1 Neuropathic pain management at discharge should rely on drugs specific for neuropathic pain, avoiding exclusive use of conventional analgesics.	100.00%	AGREEMENT
16.2 In low-back pain with radiculopathy, a multimodal approach including gabapentinoids, antidepressants, and tramadol-like agents should be considered, evaluating the personal safety profile.	100.00%	
16.3 For post-herpetic neuralgia and trigeminal neuralgia, neuropathic-specific drugs should be used already at discharge and specialist follow-up considered.	100.00%	
**Patient and caregiver education** Statement #17—“I believe that:”		
17.1 At discharge, patients and/or caregivers must receive clear written instructions on analgesic use, including dosage, timing, duration, and warning signs.	100.00%	AGREEMENT
17.2 Education should include non-pharmacological support techniques and early recognition of the need for medical re-evaluation.	100.00%	
17.3 Education should also cover recognition of warning signs or adverse events requiring return to the ED.	90.91%	

For ease of reading, thematic areas and main topics headings are reported in bold type.

**Table 6:** Final items—Thematic area 3.

THEMATIC AREA 3—ANALGESIA IN CHILDREN WITH MILD TO MODERATE PAIN		
**Pain management in pediatrics** Statement #18—“I believe that:”		
18.1 Paracetamol should be the drug of choice for moderate pain in children.	96.97%	AGREEMENT
18.2 From 3 months of age onward, ibuprofen is also a drug of choice for moderate pain.	100.00%	
18.3 The preferred route of administration for analgesia in children with moderate pain is oral or orodispersible.	100.00%	
**Multimodal therapy in children** Statement #19—“I believe that:”		
19.1 When greater pain control is needed, a fixed-dose combination of paracetamol and ibuprofen should be preferred.	100.00%	AGREEMENT
19.2 Multimodal therapy (e.g., fixed-dose paracetamol + NSAID) improves pain control and allows dose reduction of individual drugs.	100.00%	
19.3 Multimodal therapy reduces the likelihood of administration errors.	100.00%	
**Dosing in pediatric patients** Statement #20—“I believe that:”		
20.1 Paracetamol should be administered at 15 mg/kg per dose.	100.00%	AGREEMENT
20.2 Ibuprofen should be given every 8 h at 10 mg/kg for children > 6 months, 5 mg/kg for 3–6 months, up to a maximum of 1800 mg/day.	100.00%	
20.3 Analgesic therapy in children should be scheduled (rather than as needed).	100.00%	
**Opioid use in pediatrics** Statement #21—“I believe that:”		
21.1 Opioids should be used in children with moderate pain when paracetamol or NSAID monotherapy is ineffective or contraindicated.	90.91%	AGREEMENT
**21.2** Clinical monitoring must be strict when using opioids in children.	96.97%	
**21.3** Opioids should also be considered when fixed-dose paracetamol + ibuprofen is ineffective or contraindicated.	100.00%	
**Communication and adherence in children** Statement #22— “I believe that:”		
22.1 To facilitate adherence, the analgesic plan should be communicated and shared with the child and caregivers.	100.00%	AGREEMENT
22.2 Communication with the child and caregiver is essential to reduce anxiety and improve acceptance and effectiveness of therapy.	100.00%	

For ease of reading, thematic areas and main topics headings are reported in bold type.

**Table 7:** Final items—Thematic area 4.

THEMATIC AREA 4—ANALGESIA IN ELDERLY PATIENTS WITH MILD TO MODERATE PAIN		
**Pain assessment in the elderly** Statement #23—“*I believe that*:”		
23.1 In older patients, descriptive and verbal scales are most appropriate, allowing simple self-assessment even with mild-to-moderate cognitive or functional limitations.	100.00%	AGREEMENT
23.2 Recommended scales include the Numeric Rating Scale (NRS), Verbal Rating Scale (VRS), and, for severe cognitive impairment, the Pain Assessment in Advanced Dementia (PAINAD) scale.	100.00%	
**Pharmacological management in the elderly** **Statement #24**—“*I believe that*:”		
24.1 Paracetamol is the first-line treatment for moderate pain of various origins in older patients due to its favourable safety profile.	100.00%	AGREEMENT
24.2 If pain is not adequately controlled with paracetamol, adding an NSAID is an effective and safe option.	100.00%	
24.4 If pain is not controlled with paracetamol, adding oxycodone is an effective and safe option.	90.91%	
**Tailoring therapy in the elderly** Statement #25—“I believe that:”		
**25.1** Analgesic therapy should be individualized, balancing efficacy and safety and considering comorbidities and polypharmacy.	100.00%	AGREEMENT
**25.2** Potential drug interactions and dose adjustments for renal or hepatic impairment must be considered.	100.00%	
25.3 Education for elderly patients and caregivers should ensure clear communication, active adverse-event monitoring and simplified dosing to improve compliance and reduce errors, especially with cognitive or functional deficits.	100.00%	
**Fixed-dose combinations in the elderly** Statement #26—“I believe that:”		
26.1 In elderly patients with moderate pain, the use of a fixed-dose combination of analgesic drugs is preferable to monotherapy in order to increase safety, efficacy, speed and duration of the analgesic effect.	100.00%	AGREEMENT
26.2 In elderly patients, the fixed-dose combination of paracetamol with low-dose ibuprofen improves analgesic efficacy even in conditions characterized by significant inflammation.	100.00%	
26.3 In elderly patients, fixed-dose combination paracetamol/codeine administered orally is a valid treatment option for the control of moderate pain.	93.94%	
26.7 In elderly patients, fixed-dose combination paracetamol/oxycodone administered orally is a valid treatment option for the management of moderate pain.	100.00%	

For ease of reading, thematic areas and main topics headings are reported in bold type.

## Data Availability

The original contributions presented in this study are included in the article. Further inquiries can be directed to the corresponding author.

## Supplementary Materials

The following supporting information can be downloaded at: https://www.mdpi.com/article/10.3390/jcm15093230/s1, Table S1: Initial items validated by the Scientific Steering Committee; Table S2: List of panel members; Table S3: Final items; Table S4: Items in disagreement and rationale; Table S5: Excluded items and reasons; Supplementary Methods: Details of the literature review.
