# Supplementary material for: Management of Mild to Moderate Pain from Triage to Discharge in the Emergency Department: A Multidisciplinary Delphi Consensus from the Italian Society of Emergency Medicine (SIMEU)
Source: J Clin Med. 2026 Apr 23;15(9):3230. doi: 10.3390/jcm15093230 (PMC13163571; doi:10.3390/jcm15093230)
Supplement: Supplementary file 1 [file jcm-15-03230-s001.zip › jcm-4090047-supplementary.pdf]

SUPPLEMENTARY TABLES

Supplementary Table S1. Initial items validated by the Scientific Steering Committee.

Supplementary Table S2. List of panel members.

Supplementary Table S3. Final items.

Supplementary Table S4. Items in disagreement and rationale.

Supplementary Table S5. Excluded items and reasons.

Supplementary Table S1. Initial items validated by the Scientific Steering Committee.

**THEMATIC AREA 1 - ANALGESIA AT TRIAGE**
**Criteria for administering analgesics at triage**<br>**Statement #1** – *"The administration of analgesics at triage for patients with mild-to-moderate pain should be based on standardised criteria such as:"*
**1.1** *Reason for ED admission.*
**1.2** *Patient-specific characteristics.*
**Variations for vulnerable populations**<br>**Statement #2** – *"The management of analgesic therapy at triage should include specific variations for certain vulnerable populations:"*
**2.1** *Age (children and elderly).*
**2.2** *Pregnancy and breastfeeding.*
**2.3** *Specific pathologies and rare diseases.*
**Paracetamol as first-line choice**<br>**Statement #3** – *"Paracetamol should be considered the first-line choice for the pharmacological management of mild and moderate pain at triage in vulnerable populations, due to its favourable safety profile."*
**3.1** *Age (children and elderly).*
**3.2** *Pregnancy and breastfeeding.*
**3.3** *Specific pathologies and rare diseases.*
**Contraindications to be assessed**

**Statement #4** – *"The definition of a triage analgesia protocol should include the assessment of certain contraindications, such as:"*
**4.1** *Hypersensitivity or allergy to the analgesic specified in the protocol.*
**4.2** *Presence of bleeding disorders or use of anticoagulants.*
**4.3** *Concomitant use of drugs with potential interactions.*
**4.4** *Difficulty in collecting the patient's medical history.*
**Drugs appropriate for mild pain at triage**<br>**Statement #5** – *"I believe that the following drugs should be considered effective and safe for triage analgesia in patients with mild pain."*
**5.1** *Paracetamol.*
**5.2** *Ibuprofen.*
**5.3** *Fixed-dose combination of paracetamol + ibuprofen.*
**5.4** *Fixed-dose combination* of *paracetamol + codeine.*
**5.5** *Sublingual sufentanil.*
**Drugs appropriate for moderate pain at triage**<br>**Statement #6** – *"I believe that the following drugs should be considered effective and safe for triage analgesia in patients with moderate pain."*
**6.1** *Paracetamol.*
**6.2** *Ibuprofen.*
**6.3** *Fixed-dose combination of paracetamol + ibuprofen.*
**6.4** *Fixed-dose combination of paracetamol + codeine.*
**6.5** *Sublingual sufentanil.*
**Route of administration**<br>**Statement #7** – *"I believe that, based on currently available formulations:"*
**7.1** *The oral route should be considered the most advantageous for managing mild-to-moderate pain at triage due to its simplicity, safety and good efficacy.*
**7.2** *The sublingual route should be considered an alternative for managing mild pain at triage, particularly for its rapid onset of action.*

**7.3** *The sublingual route should be considered an alternative for managing moderate pain at triage, particularly for its rapid onset of action.*
**7.4** *The IV route should be considered appropriate at triage for patients with mild-to-moderate pain who cannot take oral or sublingual drugs.*
**7.5** *Alternative routes of administration should be considered valid options in selected cases within the triage management of mild-to-moderate pain.*
**Monitoring of analgesic therapy at triage**<br>**Statement #8** – *"I believe that:"*
**8.1** *The effectiveness of analgesic therapy at triage must be regularly monitored using validated pain assessment scales.*
**8.2** *Monitoring should include patient observation and re-assessment.*
**8.3** *Monitoring should also include early identification of possible adverse effects of the administered therapy.*
**8.4** *In case of partial or insufficient response to analgesic therapy at triage, pain should be re-evaluated and alternative strategies considered.*
**Organization and training**<br>**Statement #9** – *"I believe that:"*
**9.1** *Specific training of healthcare staff is a key element to be included in the organization of a triage analgesia protocol.*
**9.2** *A triage analgesia protocol should define clear treatment pathways and ensure the immediate availability of necessary analgesic drugs.*
**9.3** *Non-pharmacological approaches (e.g., immobilization, ice application) should be included in the organization of a triage analgesia protocol.*
**9.4** *ED overcrowding may represent a barrier to the implementation of the triage pain-management protocol.*
**Ethical and legal aspects**<br>**Statement #10** – *"I believe that:"*
**10.1** *Adequate pain management at triage is a patient's right and a clinical duty.*
**10.2** *Analgesia at triage should be initiated on the basis of the patient's implicit consent after adequate information.*
**10.3** *The assessment, treatment and outcome of pain management must be documented.*

THEMATIC AREA 2 - RISK FACTORS OF ANALGESIC THERAPY
**Analgesia at discharge (adult patients)**<br><br>**Statement #11** – *"I believe that:"*
**11.1** *Paracetamol should be the first-line drug at discharge for adults with mild-to-moderate pain due to its favourable safety and efficacy profile.*
**11.2** *For moderate pain with an inflammatory component, NSAIDs should be prescribed with caution in elderly patients or those at gastrointestinal, renal, or cardiovascular risk.*
**11.3** *For moderate pain in adults, paracetamol combined with an NSAID may be a valid option at discharge.*
**11.4** *Weak opioids should be considered for moderate pain at discharge, reserved for patients with contraindications to NSAIDs and/or paracetamol.*
**Drug-selection criteria at discharge**<br><br>**Statement #12** – *"I believe that:"*
**12.1** *Analgesic therapy at discharge should be guided by the assessment of individual risk factors.*
**12.2** *Concomitant therapies with potential drug interactions should guide the choice of the safest analgesic.*
**12.3** *Analgesic therapy at discharge should not be personalised according to clinical profile.*
**NSAID use**<br><br>**Statement #13** – *"I believe that:"*
**13.1** *NSAIDs should be preferred for moderate pain of musculoskeletal or post-traumatic origin.*
**13.2** *NSAIDs should be preferred for moderate pain due to renal or biliary colic.*
**13.3** *NSAIDs should be preferred for moderate pain from acute headaches (e.g., tension-type headache or migraine).*
**13.4** *The fixed-dose combination of paracetamol and ibuprofen represents a valid therapeutic option for moderate musculoskeletal or traumatic pain.*
**Opioid therapy at discharge**<br><br>**Statement #14** – *"I believe that:"*
**14.1** *Immediate-release oral oxycodone should be preferred at discharge for moderate pain, thanks to its rapid analgesic efficacy and good bioavailability.*
**14.2** *The oxycodone/paracetamol combination is indicated for moderate pain resistant to paracetamol/ibuprofen.*

**14.3** *The paracetamol/codeine combination is indicated for moderate pain when NSAIDs are contraindicated.*
**Duration of therapy at discharge**<br><br>**Statement #15** – *"I believe that:"*
**15.1** *Analgesic therapy for moderate pain at discharge should generally be prescribed for no more than 3–5 days, unless clinically indicated otherwise.*
**15.2** *A longer duration should be considered for specific conditions such as neuropathic pain.*
**15.3** *Patients should receive instructions for short-term clinical follow-up by primary care.*
**Neuropathic pain**<br><br>**Statement #16** – *"I believe that:"*
**16.1** *Neuropathic pain management at discharge should rely on drugs specific for neuropathic pain, avoiding exclusive use of conventional analgesics.*
**16.2** *In low-back pain with radiculopathy, a multimodal approach including gabapentinoids, antidepressants, and tramadol-like agents should be considered, evaluating the personal safety profile.*
**16.3** *For post-herpetic neuralgia and trigeminal neuralgia, neuropathic-specific drugs should be used already at discharge and specialist follow-up considered.*
**Patient and caregiver education**<br><br>**Statement #17** – *"I believe that:"*
**17.1** *At discharge, patients and/or caregivers must receive clear written instructions on analgesic use, including dosage, timing, duration, and warning signs.*
**17.2** *Education should include non-pharmacological support techniques and early recognition of the need for medical re-evaluation.*
**17.3** *Education should also cover recognition of warning signs or adverse events requiring return to the ED.*
**17.4** *A standardised protocol for prescribing analgesic therapy at discharge is preferable.*
**THEMATIC AREA 3 - ANALGESIA IN CHILDREN WITH MILD TO MODERATE PAIN**
**Pain management in paediatrics**<br><br>**Statement #18** – *"I believe that:"*
**18.1** *Paracetamol should be the drug of choice for moderate pain in children.*
**18.2** *From 3 months of age onward, ibuprofen is also a drug of choice for moderate pain.*

**18.3** *The preferred route of administration for analgesia in children with moderate pain is oral or orodispersible.*
**Multimodal therapy in children**<br>**Statement #19** – *"I believe that:"*
**19.1** *When greater pain control is needed, a fixed-dose combination of paracetamol and ibuprofen should be preferred.*
**19.2** *Multimodal therapy (e.g., fixed-dose paracetamol + NSAID) improves pain control and allows dose reduction of individual drugs.*
**19.3** *Multimodal therapy reduces the likelihood of administration errors.*
**Dosing in paediatric patients**<br>**Statement #20** – *"I believe that:"*
**20.1** *Paracetamol should be administered at 10–15 mg/kg per dose (up to 80 mg/kg/day).*
**20.2** *Ibuprofen should be given every 8 hours at 10 mg/kg for children >6 months, 5 mg/kg for 3–6 months, up to a maximum of 1,800 mg/day.*
**20.3** *Analgesic therapy in children should be scheduled (rather than as-needed).*
**Opioid use in paediatrics**<br>**Statement #21** – *"I believe that:"*
**21.1** *Opioids should be used in children with moderate pain when paracetamol or NSAID monotherapy is ineffective or contraindicated.*
**21.2** *Clinical monitoring must be strict when using opioids in children.*
**21.3** *Opioids should also be considered when fixed-dose paracetamol + ibuprofen is ineffective or contraindicated.*
**Communication and adherence in children**<br>**Statement #22** – *"I believe that:"*
**22.1** *To facilitate adherence, the analgesic plan should be communicated and shared with the child and caregivers.*
**22.2** *Communication with the child and caregiver is essential to reduce anxiety and improve acceptance and effectiveness of therapy.*
**THEMATIC AREA 4 - ANALGESIA IN ELDERLY PATIENTS WITH MILD TO MODERATE PAIN**
**Pain assessment in the elderly**

**Statement #23** – *"I believe that:"*
**23.1** *In older patients, descriptive and verbal scales are most appropriate, allowing simple self-assessment even with mild-to-moderate cognitive or functional limitations.*
**23.2** *Recommended scales include the Numeric Rating Scale (NRS), Verbal Rating Scale (VRS), and, for severe cognitive impairment, the Pain Assessment in Advanced Dementia (PAINAD) scale.*
**Pharmacological management in the elderly**<br>**Statement #24** – *"I believe that:"*
**24.1** *Paracetamol is the first-line treatment for moderate pain of various origins in older patients due to its favourable safety profile.*
**24.2** *If pain is not adequately controlled with paracetamol, adding an NSAID is an effective and safe option.*
**24.3** *If pain is not controlled with paracetamol, adding tramadol is an effective and safe option.*
**24.4** *If pain is not controlled with paracetamol, adding oxycodone is an effective and safe option.*
**Tailoring therapy in the elderly**<br>**Statement #25** – *"I believe that:"*
**25.1** *Analgesic therapy should be individualised, balancing efficacy and safety and considering comorbidities and polypharmacy.*
**25.2** *Potential drug interactions and dose adjustments for renal or hepatic impairment must be considered.*
**25.3** *Education for elderly patients and caregivers should ensure clear communication, active adverse-event monitoring and simplified dosing to improve compliance and reduce errors, especially with cognitive or functional deficits.*
**Fixed-dose combinations in the elderly**<br>**Statement #26** – *"I believe that:"*
**26.1** *In elderly patients with moderate pain, the use of a fixed-dose combination of analgesic drugs is preferable to monotherapy in order to increase safety, efficacy, speed and duration of the analgesic effect.*
**26.2** *In elderly patients, the fixed-dose combination of paracetamol with low-dose ibuprofen improves analgesic efficacy even in conditions characterised by significant inflammation.*
**26.3** *In elderly patients, fixed-dose combination paracetamol/codeine administered orally is a valid treatment option for the control of moderate pain.*
**26.4** *In elderly patients, fixed-dose combination paracetamol/tramadol administered orally is a valid treatment option for the control of moderate pain.*

**26.5** *In elderly patients, fixed-dose combination oral NSAIDs/codeine is a valid treatment option for the management of moderate pain.*
**26.6** *In elderly patients, fixed-dose combination oral NSAID/tramadol is a valid treatment option for the management of moderate pain*.
**26.7** *In elderly patients, fixed-dose combination paracetamol/oxycodone administered orally is a valid treatment option for the management of moderate pain.*

Supplementary Table S2. List of panel members.

**Surname**	**Name**
**Boccuzzi**	Adriana
Caporaletti	Paola
Cimmino	Claudia Sara
D’Angelo	Luciano
Epifani	Biagio
Ferrari	Rodolfo
Franzese	Paolo
Gandolfo	Enrico
Guglielmelli	Emanuele
Iorfida	Marco
Lauria	Giuseppe
Lorenzati	Bartolomeo
Malalan	Fabio
Maragno	Margherita
Molino	Giuseppe
Molle	Roberta
Nazerian	Peiman
Noto	Giovanni
Nutis	Fabio
Pelizzaro	Silvia
Petrelli	Giuseppina
Pinna Parpaglia	Paolo
Poggiali	Erika
Pontoni	Elisa
Privitera	Daniele
Rocco Pugliese	Francesco
Ricciardelli	Adelina
Saggese	Maria Paola

Savioli	Gabriele
Silla	Aldo
Michele Domenico	Spampinato
Susi	Beniamino
Voza	Antonio

Supplementary Table S3. Final items.

**Statement**<br>**Item**	**Percentage of panellists' responses**			**Results**	**References/comments**
	**1 - 2 (Strongly disagree - Disagree)**	**3 (Uncertain)**	**4 - 5 (Mostly agree - Strongly agree)**		
**THEMATIC AREA 1 - ANALGESIA AT TRIAGE**					
**Criteria for administering analgesics at triage**<br>**Statement #1** – *"The administration of analgesics at triage for patients with mild-to-moderate pain should be based on standardised criteria such as:"*					
**1.1** *Reason for ED admission*	0.00%	0.00%	100.00%	AGREEMENT	Fabbri et al., 2023; Rech et al., 2022; Larsson et al., 2025; SIAARTI et al., 2023.
**1.2** *Patient-specific characteristics*	0.00%	0.00%	100.00%	AGREEMENT	
**Variations for vulnerable populations**<br>**Statement #2** – *"The management of analgesic therapy at triage should include specific variations for certain vulnerable populations:"*					
**2.1** *Age (children and elderly)*	3.03%	0.00%	96.97%	AGREEMENT	Pickering et al., 2024; Elder et al., 2023; Parri et al., 2023; SIAARTI et al., 2023.
**2.2** *Pregnancy and breastfeeding*	3.03%	0.00%	96.97%	AGREEMENT	

**2.3** *Specific pathologies and rare diseases*	21.21%	3.03%	75.76%	AGREEMENT	Pickering et al., 2024; Elder et al., 2023; Parri et al., 2023; SIAARTI et al., 2023.<br><br>Vulnerable populations include patients at the extremes of age (children and older adults), those with specific physiological conditions (pregnancy or lactation), and individuals with particular or rare diseases. Growing awareness within emergency triage systems has led to the development of dedicated pathways for patients with special or rare conditions - for example, those with spinal muscular atrophy (SMA) - often in collaboration with family physicians and caregivers. Scientific societies are promoting structured care

					protocols for these groups. In pain management, non-pharmacological interventions and procedural adaptations are also relevant. However, consensus on this statement was lower, as panellists considered that triage differentiation based on rare conditions is still difficult to apply in daily clinical practice.
**Paracetamol as first-line choice**<br>**Statement #3** – *"Paracetamol should be considered the first-line choice for the pharmacological management of mild and moderate pain at triage in vulnerable populations, due to its favorable safety profile."*					
**3.1** *Age (children and elderly)*	0.00%	3.03%	96.97%	AGREEMENT	Freo et al., 2021; Scaglione, 2022; SIAARTI et al., 2023.
**3.2** *Pregnancy and breastfeeding*	0.00%	0.00%	100.00%	AGREEMENT	
**3.3** *Specific pathologies and rare diseases*	0.00%	21.21%	78.79%	AGREEMENT	Freo et al., 2021; Scaglione, 2022; SIAARTI et al., 2023.<br><br>In the first Delphi round, consensus was not achieved because

					this category includes a wide and heterogeneous spectrum of conditions, and some clinicians suggested that alternative approaches might be needed for specific cases. During the second round, agreement was reached, reaffirming that mild-to-moderate pain in patients with special or rare diseases should always be treated at triage. Paracetamol was confirmed as the safest first-line option in most of these conditions, consistently supported by current evidence.
**Contraindications to be assessed**<br>**Statement #4** – *"The definition of a triage analgesia protocol should include the assessment of certain contraindications, such as:"*					
**4.1** *Hypersensitivity or allergy to the analgesic specified in the protocol*	0.00%	0.00%	100.00%	AGREEMENT	Hersh et al., 2007; Toes et al., 2005; Brune and Patrignani, 2015;

**4.2** *Presence of bleeding disorders or use of anticoagulants*	0.00%	0.00%	100.00%	AGREEMENT	Bindu et al., 2020; SIAARTI et al., 2023.
**4.3** *Concomitant use of drugs with potential interactions*	0.00%	6.06%	93.94%	AGREEMENT	
**4.4** *Difficulty in collecting the patient's medical history*	0.00%	0.00%	100.00%	AGREEMENT	
**Drugs appropriate for mild pain at triage**<br>**Statement #5** – *"I believe that the following drugs are appropriate, in terms of risk/benefit ratio, for analgesia at triage in patients with mild pain."*					The original Statement 5 "*I believe that the following drugs should be considered effective and safe for triage analgesia in patients with mild pain*" was reformulated to emphasise the importance of the benefit–risk balance.
**5.1** *Paracetamol*	0.00%	0.00%	100.00%	AGREEMENT	Freo et al., 2021; Scaglione, 2022; Brune and Patrignani, 2015; Bindu et al., 2020.
**5.2** *Ibuprofen*	0.00%	9.09%	90.91%	AGREEMENT	
**Drugs appropriate for moderate pain at triage**<br>**Statement #6** – *"I believe that the following drugs are appropriate, in terms of risk/benefit ratio, for analgesia at triage in patients with moderate pain."*					The original Statement 6 "*I believe that the following drugs should be considered effective and safe for triage*

					analgesia in patients with moderate pain" was reformulated to emphasise the importance of the benefit–risk balance.
**6.1** *Paracetamol*	0.00%	0.00%	100.00%	AGREEMENT	Freo et al., 2021; Brune and Patrignani, 2015; Bindu et al., 2020.
**6.2** *Ibuprofen*	0.00%	0.00%	100.00%	AGREEMENT	
**6.3** *Fixed-dose combination of paracetamol + ibuprofen*	0.00%	0.00%	100.00%	AGREEMENT	Bettiol et al., 2021; Derry et al., 2013; Rech et al., 2022.
**6.4** *Fixed-dose combination of paracetamol + codeine*	12.12%	6.06%	81.82%	AGREEMENT	Franceschi et al., 2013; Fornasari and Lora Aprile, 2018.
**6.5** *Sublingual sufentanil*	6.06%	12.12%	81.82%	AGREEMENT	SIAARTI et al., 2023; Rech et al., 2022<br><br>Sublingual sufentanil is indicated for moderate pain and, according to the summary of product characteristics, is currently the only opioid that can be administered by nurses in the emergency setting. Its strategic

					role at triage was emphasised. Lack of consensus in early rounds was mainly attributed to its limited availability and high cost. Some panellists highlighted the need for accurate administration, with careful evaluation of moderate pain intensity (NRS 4–6). At third round, the panel agreed that sublingual sufentanil should be considered an option for selected emergency departments, depending on the type and volume of patient inflow. Its inclusion underscores the importance of maintaining opioid alternatives for moderate pain when non-opioid or oral options are unsuitable.
**Route of administration**<br><br>**Statement #7** – *"I believe that, based on currently available formulations:"*					

**7.1** *The oral route should be considered the most advantageous for managing mild-to-moderate pain at triage due to its simplicity, safety and good efficacy.*	0.00%	0.00%	100.00%	AGREEMENT	Rech et al., 2022; Fabbri et al., 2023; Miner et al., 2008; SIAARTI et al., 2023.
**7.3** *The sublingual route should be considered an alternative for managing moderate pain at triage, particularly for its rapid onset of action.*	3.03%	3.03%	93.94%	AGREEMENT	Rech et al., 2022; Fabbri et al., 2023; SIAARTI et al., 2023<br><br>The sublingual route was considered appropriate for the management of moderate pain at triage. Panellists recognised its advantages—rapid absorption, ease of administration, and suitability for patients unable to take oral medication. Sublingual sufentanil, in particular, was highlighted as a valuable option for nurse-administered analgesia in selected EDs, provided that

					staff are adequately trained and local protocols are in place (*see also 6.5 and 7.2*).
**7.4** *The IV route should be considered appropriate at triage for patients with mild-to-moderate pain who cannot take oral or sublingual drugs.*	0.00%	0.00%	100.00%	AGREEMENT	SIAARTI et al., 2023; Fabbri et al., 2023; Miner et al., 2008.
**7.5** *Alternative routes of administration should be considered valid options in selected cases within the triage management of mild-to-moderate pain.*	0.00%	3.03%	96.97%	AGREEMENT	Parri et al., 2023; Castagno et al., 2024; SIAARTI et al., 2023.<br><br>Among alternative routes of administration, the intranasal and orodispersible formulations are the most frequently used, particularly in paediatric patients, while the intramuscular route is rarely employed at triage. These routes are considered valid options for selected cases of mild-to-

					moderate pain when they improve feasibility, comfort, or patient cooperation.
**Monitoring of analgesic therapy at triage**<br>**Statement #8** – *"I believe that:"*					
**8.1** *The effectiveness of analgesic therapy at triage must be regularly monitored using validated pain assessment scales.*	0.00%	0.00%	100.00%	AGREEMENT	Rech et al., 2022; Fabbri et al., 2023; Larsson et al., 2025; SIAARTI et al., 2023
**8.2** *Monitoring should include patient observation and re-assessment.*	0.00%	0.00%	100.00%	AGREEMENT	
**8.3** *Monitoring should also include early identification of possible adverse effects of the administered therapy.*	0.00%	0.00%	100.00%	AGREEMENT	
**8.4** *In case of partial or insufficient response to analgesic therapy at triage, pain should be re-evaluated and alternative strategies considered.*	9.09%	9.09%	81.82%	AGREEMENT	Rech et al., 2022; Fabbri et al., 2023; Larsson et al., 2025; SIAARTI et al., 2023.<br><br>Patient reassessment following analgesic therapy is an integral

					part of triage protocols. However, clinical practices vary regarding the extent and timing of monitoring, even in cases of mild pain. Differences were also noted in how to manage partial response—whether to add another analgesic or adopt alternative strategies. The panel agreed on the importance of systematic pain re-evaluation and highlighted the need for clearer guidance on subsequent therapeutic steps to ensure consistency across EDs.
**Organization and training**<br>**Statement #9** – *"I believe that:"*					
**9.1** *Specific training of healthcare staff is a key element to be included in the organization of a*	0.00%	0.00%	100.00%	AGREEMENT	Fabbri et al., 2023; Larsson et al., 2025; SIAARTI et al., 2023.

triage analgesia protocol.					
**9.2** *A triage analgesia protocol should define clear treatment pathways and ensure the immediate availability of necessary analgesic drugs.*	0.00%	0.00%	100.00%	AGREEMENT	SIAARTI et al., 2023; Rech et al., 2022.
**9.3** *Non-pharmacological approaches (e.g., immobilization, ice application) should be included in the organization of a triage analgesia protocol.*	0.00%	0.00%	100.00%	AGREEMENT	Fabbri et al., 2023; SIAARTI et al., 2023.
**Ethical and legal aspects** **Statement #10** – *"I believe that:"*					
**10.1** *Adequate pain management at triage is a patient's right and a clinical duty.*	0.00%	0.00%	100.00%	AGREEMENT	SIAARTI et al., 2023; Zanza et al., 2023.
**10.2** *Analgesia at triage should be initiated on the basis of the patient's implicit consent after adequate information.*	0.00%	0.00%	100.00%	AGREEMENT	

**10.3** *The assessment, treatment and outcome of pain management must be documented.*	0.00%	0.00%	100.00%	AGREEMENT	SIAARTI et al., 2023; Fabbri et al., 2023.
**THEMATIC AREA 2 - RISK FACTORS OF ANALGESIC THERAPY**					
**Analgesia at discharge (adult patients)**<br>**Statement #11** – *"I believe that:"*					
**11.1** *Paracetamol should be the first-line drug at discharge for adults with mild-to-moderate pain due to its favourable safety and efficacy profile.*	0.00%	0.00%	100.00%	AGREEMENT	Alchin et al., 2022; Freo et al., 2021; Scaglione, 2022; SIAARTI et al., 2023.
**11.2** *For moderate pain with an inflammatory component, NSAIDs should be prescribed with caution in elderly patients or those at gastrointestinal, renal, or cardiovascular risk.*	0.00%	0.00%	100.00%	AGREEMENT	Brune and Patrignani, 2015; Bindu et al., 2020; SIAARTI et al., 2023.
**11.3** *For moderate pain in adults, paracetamol combined with an NSAID may be a valid option at discharge.*	0.00%	3.03%	96.97%	AGREEMENT	Bettiol et al., 2021; Derry et al., 2013; Rech et al., 2022.
**11.4** *Weak opioids should be considered for moderate pain at*	0.00%	3.03%	96.97%	AGREEMENT	Alchin et al., 2022; Freo et al., 2021; Scaglione, 2022.

discharge, reserved for patients with contraindications to NSAIDs and/or paracetamol.					
**Drug-selection criteria at discharge**<br>**Statement #12** – *"I believe that:"*					
**12.1** *Analgesic therapy at discharge should be guided by the assessment of individual risk factors.*	0.00%	0.00%	100.00%	AGREEMENT	Alchin et al., 2022; Brune and Patrignani, 2015; Bindu et al., 2020.
**12.2** *Concomitant therapies with potential drug interactions should guide the choice of the safest analgesic.*	0.00%	0.00%	100.00%	AGREEMENT	Brune and Patrignani, 2015; Bindu et al., 2020; Chatterjee et al., 2015; SIAARTI et al., 2023.
**NSAID use**<br>**Statement #13** – *"I believe that:"*					
**13.2** *NSAIDs should be preferred for moderate pain due to renal or biliary colic.*	0.00%	0.00%	100.00%	AGREEMENT	Brune and Patrignani, 2015; Bindu et al., 2020; SIAARTI et al., 2023.
**13.3** *NSAIDs should be preferred for moderate pain from acute headaches (e.g., tension-type headache or migraine).*	0.00%	0.00%	100.00%	AGREEMENT	Brune and Patrignani, 2015; Bindu et al., 2020; Alchin et al., 2022.

**13.4** *The fixed-dose combination of paracetamol and ibuprofen represents a valid therapeutic option for moderate musculoskeletal or traumatic pain.*	0.00%	0.00%	100.00%	AGREEMENT	Bettiol et al., 2021; Derry et al., 2013; Rech et al., 2022.
**Opioid therapy at discharge** **Statement #14** – *"I believe that:"*					
**14.2** *The oxycodone/paracetamol combination is indicated for moderate pain resistant to paracetamol/ibuprofen.*	3.03%	9.09%	87.88%	AGREEMENT	Natoli et al., 2016; Manassero and Bossolasco, 2021; SIAARTI et al., 2023.
**Duration of therapy at discharge** **Statement #15** – *"I believe that:"*					
**15.1** *Analgesic therapy for moderate pain at discharge should generally be prescribed for no more than 3–5 days, unless clinically indicated otherwise.*	0.00%	0.00%	100.00%	AGREEMENT	SIAARTI et al., 2023; Rech et al., 2022.
**15.2** *A longer duration should be considered for specific conditions such as neuropathic pain.*	0.00%	0.00%	100.00%	AGREEMENT	Doneddu et al., 2023; SIAARTI et al., 2023.

**15.3** *Patients should receive instructions for short-term clinical follow-up by primary care.*	0.00%	0.00%	100.00%	AGREEMENT	Rech et al., 2022; Chi et al., 2020; SIAARTI et al., 2023.
**Neuropathic pain**<br>**Statement #16** – *"I believe that:"*					
**16.1** *Neuropathic pain management at discharge should rely on drugs specific for neuropathic pain, avoiding exclusive use of conventional analgesics.*	0.00%	0.00%	100.00%	AGREEMENT	Doneddu et al., 2023; Chi et al., 2020; Lecomte et al., 2011; SIAARTI et al., 2023.<br><br>For patients presenting with neuropathic pain, the panel emphasised the need to prescribe agents specifically indicated for neuropathic mechanisms rather than relying solely on conventional analgesics. Although discharge management is often handled by general practitioners, the Delphi panel strongly recommended specialist follow-up to ensure appropriate diagnosis, titration, and

					long-term monitoring of therapy.
**16.2** *In low-back pain with radiculopathy, a multimodal approach including gabapentinoids, antidepressants, and tramadol-like agents should be considered.*	0.00%	0.00%	100.00%	AGREEMENT	Doneddu et al., 2023; Chi et al., 2020; SIAARTI et al., 2023.
**16.3** *For post-herpetic neuralgia and trigeminal neuralgia, neuropathic-specific drugs should be used already at discharge and specialist follow-up considered.*	0.00%	0.00%	100.00%	AGREEMENT	
**Patient and caregiver education**<br>**Statement #17** – *"I believe that:"*					
**17.1** *At discharge, patients and/or caregivers must receive clear written instructions on analgesic use, including dosage, timing, duration, and warning signs.*	0.00%	0.00%	100.00%	AGREEMENT	Chi et al., 2020; SIAARTI et al., 2023.

**17.2** *Education should include non-pharmacological support techniques and early recognition of the need for medical re-evaluation.*	0.00%	0.00%	100.00%	AGREEMENT	
**17.3** *Education should also cover recognition of warning signs or adverse events requiring return to the ED.*	0.00%	9.09%	90.91%	AGREEMENT	
**THEMATIC AREA 3 - ANALGESIA IN CHILDREN WITH MILD TO MODERATE PAIN**					
**Pain management in paediatrics** **Statement #18** – *"I believe that:"*					
**18.1** *Paracetamol should be the drug of choice for moderate pain in children.*	0.00%	3.03%	96.97%	AGREEMENT	Benini et al., 2020; Bailey and Trottier, 2016; Thibault et al., 2023; SIAARTI et al., 2023; Ceelie et al., 2013.
**18.2** *From 3 months of age onward, ibuprofen is also a drug of choice for moderate pain.*	0.00%	0.00%	100.00%	AGREEMENT	Bailey and Trottier, 2016; Pickering et al., 2002; SIAARTI et al., 2023; Ceelie et al., 2013.
**18.3** *The preferred route of administration for analgesia in children*	0.00%	0.00%	100.00%	AGREEMENT	Parri et al., 2023; Thibault et al., 2023; SIAARTI et al., 2023.

with moderate pain is oral or orodispersible.					The panel agreed that the oral or orodispersible routes are the preferred options for managing moderate pain in children, as they are effective, practical, and cost-efficient. While no statement was made about other formulations such as rectal or intravenous, it was noted that these are sometimes used in paediatric settings, particularly when oral administration is not feasible. The challenge of choosing routes in children versus adults - where intravenous administration is more readily available - was highlighted. The orodispersible route was specifically defined as "preferred" due to its advantages in

					terms of evidence, cost, and practicality.
**Multimodal therapy in children**<br>**Statement #19** – *"I believe that:"*					
**19.1** *When greater pain control is needed, a fixed-dose combination of paracetamol and ibuprofen should be preferred.*	0.00%	0.00%	100.00%	AGREEMENT	Parri et al., 2023; Castagno et al., 2024; Benini et al., 2020; Ceelie et al., 2013.<br><br>The panel strongly agreed that, when greater pain control is needed, a fixed-dose combination of paracetamol and ibuprofen should be preferred over monotherapy. This combination offers synergistic effects, improving pain relief while reducing the required dose of each drug. Multimodal therapy is particularly recommended when monotherapy fails, as it allows for a broader range of strategies in managing moderate pain. The moderate

					pain range, which spans from mild to severe, permits flexibility in selecting the most appropriate treatment approach based on patient needs and clinical context.
**19.2** *Multimodal therapy (e.g., fixed-dose paracetamol + NSAID) improves pain control and allows dose reduction of individual drugs.*	0.00%	0.00%	100.00%	AGREEMENT	Parri et al., 2023; Castagno et al., 2024; Ceelie et al., 2013.
**19.3** *Multimodal therapy reduces the likelihood of administration errors.*	0.00%	0.00%	100.00%	AGREEMENT	Parri et al., 2023; Castagno et al., 2024; Bailey and Trottier, 2016; Ceelie et al., 2013.
**Dosing in paediatric patients**<br>**Statement #20** – *"I believe that:"*					
**20.1** *Paracetamol should be administered at 15 mg/kg per dose.*	0.00%	0.00%	100.00%	AGREEMENT	Thibault et al., 2023; Bailey and Trottier, 2016; SIAARTI et al., 2023; Ceelie et al., 2013.<br><br>The panel discussed paracetamol dosing in

					detail, agreeing on the dosage of 15 mg/kg per dose. The posology from the Summary of Product Characteristics (SmPC) varies for the different paracetamol formulations, with oral suspension typically prescribed for 4 doses per day (every 5-6 hours) and other formulations allowing for up to 8 doses per day (every 3-4 hours).
**20.2** *Ibuprofen should be given every 8 hours at 10 mg/kg for children >6 months, 5 mg/kg for 3–6 months, up to a maximum of 1,800 mg/day.*	0.00%	0.00%	100.00%	AGREEMENT	Thibault et al., 2023; Bailey and Trottier, 2016; SIAARTI et al., 2023; Ceelie et al., 2013.<br><br>For ibuprofen, specific dosing recommendations based on age and weight were discussed. For children over 6 months, 10 mg/kg should be administered every 8 hours, while for 3–6 months, the

					dose should be reduced to 5 mg/kg every 8 hours, up to a maximum of 1,800 mg/day. This dosing schedule ensures both efficacy and safety while minimizing the risk of overdose.
**20.3** *Analgesic therapy in children should be scheduled (rather than as-needed).*	0.00%	0.00%	100.00%	AGREEMENT	Bailey and Trottier, 2016; SIAARTI et al., 2023; Ceelie et al., 2013.<br><br>The panel emphasised the importance of scheduled analgesic therapy for children, rather than administering pain relief on an as-needed basis. Scheduled administration ensures continuous pain control, helps maintain stable plasma concentrations, and improves overall patient comfort. This approach should be prioritised to prevent

					undertreatment and to provide effective, consistent analgesia.
**Opioid use in paediatrics**<br>**Statement #21** – *"I believe that:"*					
**21.1** *Opioids should be used in children with moderate pain when paracetamol or NSAID monotherapy is ineffective or contraindicated.*	0.00%	9.09%	90.91%	AGREEMENT	Bailey and Trottier, 2016; SIAARTI et al., 2023.
**21.2** *Clinical monitoring must be strict when using opioids in children.*	0.00%	3.03%	96.97%	AGREEMENT	Bailey and Trottier, 2016; Benini et al., 2020; SIAARTI et al., 2023.
**21.3** *Opioids should also be considered when fixed-dose paracetamol + ibuprofen is ineffective or contraindicated.*	0.00%	0.00%	100.00%	AGREEMENT	Bailey and Trottier, 2016; Thibault et al., 2023; SIAARTI et al., 2023.
**Communication and adherence in children**<br>**Statement #22** – *"I believe that:"*					
**22.1** *To facilitate adherence, the analgesic plan should be communicated and shared with the child and caregivers.*	0.00%	0.00%	100.00%	AGREEMENT	Benini et al., 2020; Bailey and Trottier, 2016; SIAARTI et al., 2023.

**22.2** *Communication with the child and caregiver is essential to reduce anxiety and improve acceptance and effectiveness of therapy.*	0.00%	0.00%	100.00%	AGREEMENT	
**THEMATIC AREA 4 - ANALGESIA IN ELDERLY PATIENTS WITH MILD TO MODERATE PAIN**					
**Pain assessment in the elderly**<br>**Statement #23** – *"I believe that:"*					
**23.1** *In older patients, descriptive and verbal scales are most appropriate, allowing simple self-assessment even with mild-to-moderate cognitive or functional limitations.*	0.00%	0.00%	100.00%	AGREEMENT	Casey and Niznik, 2025; Terrell et al., 2009; Elder et al., 2023
**23.2** *Recommended scales include the Numeric Rating Scale (NRS), Verbal Rating Scale (VRS), and, for severe cognitive impairment, the Pain Assessment in Advanced Dementia (PAINAD) scale.*	0.00%	0.00%	100.00%	AGREEMENT	
**Pharmacological management in the elderly**<br>**Statement #24** – *"I believe that:"*					

**24.1** *Paracetamol is the first-line treatment for moderate pain of various origins in older patients due to its favourable safety profile.*	0.00%	0.00%	100.00%	AGREEMENT	Freo et al., 2021; Pickering et al., 2024; Elder et al., 2023; Casey and Niznik, 2025; Hwang and Platts-Mills, 2013; Viallon et al., 2007.
**24.2** *If pain is not adequately controlled with paracetamol, adding an NSAID is an effective and safe option.*	0.00%	0.00%	100.00%	AGREEMENT	Brune and Patrignani, 2015; Pickering et al., 2024; Elder et al., 2023; Casey and Niznik, 2025; Hwang and Platts-Mills, 2013.
**24.4** *If pain is not controlled with paracetamol, adding oxycodone is an effective and safe option.*	3.03%	6.06%	90.91%	AGREEMENT	Natoli et al., 2016; Pickering et al., 2024; Elder et al., 2023.
**Tailoring therapy in the elderly**<br>**Statement #25** – *"I believe that:"*					
**25.1** *Analgesic therapy should be individualised, balancing efficacy and safety and considering comorbidities and polypharmacy.*	0.00%	0.00%	100.00%	AGREEMENT	Elder et al., 2023; Pickering et al., 2024; Casey and Niznik, 2025.
**25.2** *Potential drug interactions and dose*	0.00%	0.00%	100.00%	AGREEMENT	

adjustments for renal or hepatic impairment must be considered.					
**25.3** *Education for elderly patients and caregivers should ensure clear communication, active adverse-event monitoring and simplified dosing to improve compliance and reduce errors, especially with cognitive or functional deficits.*	0.00%	0.00%	100.00%	AGREEMENT	Terrell et al., 2009; Elder et al., 2023; Pickering et al., 2024; Casey and Niznik, 2025.
**Fixed-dose combinations in the elderly**<br>**Statement #26** – *"I believe that:"*					
**26.1** *In elderly patients with moderate pain, the use of a fixed-dose combination of analgesic drugs is preferable to monotherapy in order to increase safety, efficacy, speed and duration of the analgesic effect.*	0.00%	0.00%	100.00%	AGREEMENT	Pickering et al., 2024; Elder et al., 2023; Casey and Niznik, 2025.
**26.2** *In elderly patients, the fixed-dose*	0.00%	0.00%	100.00%	AGREEMENT	Bettiol et al., 2021; Pickering et al., 2024;

combination of paracetamol with low-dose ibuprofen improves analgesic efficacy even in conditions characterised by significant inflammation.					Casey and Niznik, 2025; Viallon et al., 2007.
**26.3** *In elderly patients, fixed-dose combination paracetamol/codeine administered orally is a valid treatment option for the control of moderate pain.*	0.00%	6.06%	93.94%	AGREEMENT	Natoli et al., 2016; Pickering et al., 2024; Casey and Niznik, 2025.
**26.7** *In elderly patients, fixed-dose combination paracetamol/oxycodone administered orally is a valid treatment option for the management of moderate pain.*	0.00%	0.00%	100.00%	AGREEMENT	Natoli et al., 2016; Pickering et al., 2024; Casey and Niznik, 2025.

Supplementary Table S4. Items in disagreement and rationale.

**Statement Item**	**Percentage of panellist's responses**			**Results**	**References/comments**
	**1 – 2 (Strongly disagree**	**3 (Uncertain)**	**4 – 5 (Mostly agree –**		

	**– Disagree)**		**Strongly agree)**		
**THEMATIC AREA 1 - ANALGESIA AT TRIAGE**					
**Drugs appropriate for mild pain at triage**<br>**Statement #5** – *"I believe that the following drugs are appropriate, in terms of risk/benefit ratio, for analgesia at triage in patients with mild pain."*					
**5.3** *Fixed-dose combination of paracetamol + ibuprofen*	75.76%	24.24%	0.00%	DISAGREEMENT	Bettiol et al., 2021; Derry et al., 2013; Rech et al., 2022.<br><br>In the first Delphi round, consensus was not reached because some panellists considered that the fixed-dose combination of paracetamol and ibuprofen, although effective and safe, is not indicated for mild pain. After rewording, the item reached disagreement in the second round, as the panel judged its use in this context to represent overtreatment, with an

					unfavourable risk–benefit ratio.
**5.4** *Fixed-dose combination of paracetamol + codeine*	96.97%	3.03%	0.00%	DISAGREEMENT	Franceschi et al., 2013; Fornasari and Lora Aprile, 2018.<br><br>In the first round, several panellists expressed concerns regarding the use of the FDC of paracetamol and codeine for mild pain, due to the risk of side effects (which may include nausea, vomiting, allergic reactions, drowsiness, respiratory depression and other gastrointestinal and skin disorders) and the limited additional benefit over paracetamol alone. After rewording, the panel reached disagreement, confirming that this combination should not be used at triage

					for mild pain, where safer non-opioid options are sufficient.
**5.5** *Sublingual sufentanil*	100.00%	0.00%	0.00%	DISAGREEMENT	Rech et al., 2022; SIAARTI et al., 2023.<br><br>The use of sublingual sufentanil for mild pain did not reach consensus and was ultimately rejected. Although the drug is considered effective and rapidly acting, panellists agreed it should not be used for mild pain at triage because it represents an unnecessary exposure to an opioid. The latest Italian guidelines and expert webinars suggest a cautious approach to the use of sublingual sufentanil, supporting its use in specific contexts, especially postoperative or moderate-to-severe pain, while

					discouraging its use for mild pain at triage to limit unnecessary opioid exposure, consistent with the summary stating its rejection for mild pain. Its role was recognised only in selected cases of moderate pain where nurse-administered options are limited.
**Route of administration**<br><br>**Statement #7** – *"I believe that, based on currently available formulations:"*					
**7.2** *The sublingual route should be considered an alternative for managing mild pain at triage, particularly for its rapid onset of action.*	66.67%	3.03%	30.30%	DISAGREEMEN T	Rech et al., 2022; SIAARTI et al., 2023.<br><br>The use of the sublingual route for mild pain did not reach consensus. Although sublingual formulations ensure a rapid onset, panellists agreed that they are unnecessary in mild cases, where oral administration is safe, effective, and sufficient. The use of

					sublingual analgesia at triage was therefore considered inappropriate for this pain level (*see also 6.5 and 7.3*).
**THEMATIC AREA 2 - RISK FACTORS OF ANALGESIC THERAPY**					
**Drug-selection criteria at discharge**<br>**Statement #12** – *"I believe that:"*					
**12.3** *Analgesic therapy at discharge should not be personalised according to clinical profile.*	100.00%	0.00%	0.00%	DISAGREEMENT	Brune and Patrignani, 2015; Elder et al., 2023; Chatterjee et al., 2015; Pickering et al., 2024.<br><br>The item addressed the importance of evaluating polytherapy and potential drug interactions when selecting an analgesic at discharge. Panellists agreed that older or patients in polytherapy are at higher risk of adverse events, particularly when NSAIDs are added to existing

					treatments. The discussion also highlighted the need for deprescribing unnecessary medications and promoting the shortest effective analgesic regimens. The consensus reinforced that prescribing decisions should balance efficacy and safety, following a patient-centred and risk-based approach.
**NSAID use**<br><br>**Statement #13** – *"I believe that:"*					
**13.1** *NSAIDs should be preferred for moderate pain of musculoskeletal or post-traumatic origin.*	81.82%	18.18%	0.00%	DISAGREEMENT	Bettiol et al., 2021; Rech et al., 2022; Brune and Patrignani, 2015.<br><br>In the first round, panellists expressed mixed opinions regarding the systematic use of NSAIDs for musculoskeletal pain. Although NSAIDs are

					effective anti-inflammatory agents, several experts emphasised their risk of adverse effects and the lack of clear superiority over paracetamol for mild-to-moderate musculoskeletal pain. During the second round, disagreement was confirmed, as the panel favoured an individualised, risk-based approach over a blanket preference for NSAIDs.
**Opioid therapy at discharge**<br><br>**Statement #14** – *"I believe that:"*					
**14.1** *Immediate-release oral oxycodone should be preferred at discharge for moderate pain, thanks to its rapid analgesic efficacy and good bioavailability.*	66.67%	0.00%	33.33%	DISAGREEMENT	Manassero and Bossolasco, 2021; Natoli et al., 2016; SIAARTI et al., 2023.<br><br>This item did not reach consensus in the first Delphi round, as opinions diverged on the role of immediate-release oxycodone at

					discharge. Although oxycodone is effective in moderate pain, the panel highlighted concerns regarding the risk of overuse, dependence, and adverse events, particularly when prescribed without reassessment. In the second round, disagreement was maintained, and the panel did not endorse the routine use of immediate-release oxycodone at discharge, recommending instead cautious, short-term administration within a multimodal, individualised strategy.
**Patient and caregiver education**<br>**Statement #17** – *"I believe that:"*					
**17.4** *A standardised protocol for prescribing analgesic*	100.00%	0.00%	0.00%	DISAGREEMENT	SIAARTI et al., 2023.<br><br>Panellists agreed on the importance of

therapy at discharge is preferable.					structured guidance. However, there was disagreement on the feasibility of implementing a universally standardised discharge protocol. Experts emphasised that discharge plans must remain flexible, with tailored strategies based on patient-specific needs, medical history, and the complexity of pain management. Thus, the consensus supported a more individualised approach, allowing local adaptations and professional judgment.
**THEMATIC AREA 4 - ANALGESIA IN ELDERLY PATIENTS WITH MILD TO MODERATE PAIN**					
**Pharmacological management in the elderly**<br>**Statement #24** – *"I believe that:"*					
**24.3** *If pain is not controlled with paracetamol, adding*	78.79%	21.21%	0.00%	DISAGREEMENT	Häuser et al., 2021; Elder et al., 2023; Pickering et al., 2024.

tramadol is an effective and safe option.					The panel disagreed on the addition of tramadol when paracetamol alone fails to provide adequate pain relief. Some experts proposed tramadol as an add-on to paracetamol monotherapy, while others suggested combining both drugs from the outset. The discussion clarified that combining paracetamol and codeine is not recommended if the maximum dose of 1.5 g/day of paracetamol has already been reached. Therefore, codeine was excluded from the debate as an add-on option. In contrast, tramadol may be considered as an alternative, but with caution due to its

					side effect profile and the risk of sedation.
**Fixed-dose combinations in the elderly**<br>**Statement #26** – *"I believe that:"*					
**26.4** *In elderly patients, fixed-dose combination paracetamol/tramadol administered orally is a valid treatment option for the control of moderate pain.*	90.91%	9.09%	0.00%	DISAGREEMENT	Häuser et al., 2021; Pickering et al., 2024; Casey and Niznik, 2025; Hwang and Platts-Mills, 2013.<br><br>There was disagreement regarding the use of paracetamol + tramadol in elderly patients for moderate pain management. While FDC of paracetamol and tramadol are available, they are not commonly used in triage settings. Some experts raised concerns about the risk of sedation and adverse events, especially in frail elderly patients. Despite its potential effectiveness, the

					panel noted that this combination is rarely employed in clinical practice due to these risks.
**26.5** *In elderly patients, fixed-dose combination oral NSAIDs/codeine is a valid treatment option for the management of moderate pain.*	84.85%	15.15%	0.00%	DISAGREEMEN T	Brune and Patrignani, 2015; Häuser et al., 2021; Casey and Niznik, 2025; Hwang and Platts-Mills, 2013.<br><br>The panel disagreed on the use of oral NSAID + codeine in elderly patients with moderate pain. While this combination is effective for pain relief, concerns about gastrointestinal and renal side effects, especially in the elderly population, led to its exclusion from routine triage protocols. The consensus emphasised that NSAIDs combined with opioids should be carefully considered

					and prescribed only when other analgesics fail to provide adequate relief.
**26.6** *In elderly patients, fixed-dose combination oral NSAID/tramadol is a valid treatment option for the management of moderate pain.*	96.97%	3.03%	0.00%	DISAGREEMENT	Brune and Patrignani, 2015; Häuser et al., 2021; Casey and Niznik, 2025; Hwang and Platts-Mills, 2013.<br><br>The use of oral NSAID + tramadol for moderate pain in elderly patients was also met with disagreement. While this combination can offer pain relief, its use is controversial due to the potential for adverse effects such as sedation and gastrointestinal complications. Panellists agreed that while effective, this combination should be approached with caution, especially in frail or elderly patients in polytherapy. As

					with other opioid combinations, its use should be reserved for specific cases where alternative treatments are not effective.

Supplementary Table S5. Excluded items and reasons.

**Statement**<br><br>**Item**	**Percentage of panellists response**			**Results**	**References/comments**
	**1 – 2 (Strongly disagree – Disagree)**	**3 (Uncertain)**	**4 – 5 (Mostly agree – Strongly agree)**		
**THEMATIC AREA 1- ANALGESIA AT TRIAGE**					
**Organization and training**<br><br>**Statement #9** – *"I believe that:"*					
**9.4** *ED overcrowding may represent a barrier to the implementation of the triage pain-management protocol.*	66.67%	8.33%	25.00%	ELIMINATED	Pines and Hollander, 2008; Pines et al., 2010.<br><br>Panellists agreed that while these organizational challenges can negatively affect the timeliness and quality of analgesic administration, they cannot ethically justify undertreatment of pain. The item was deleted.
**THEMATIC AREA 2 - RISK FACTORS OF ANALGESIC THERAPY**					
**Opioid therapy at discharge**<br><br>**Statement #14** – *"I believe that:"*					
**14.3** *The paracetamol/codeine combination is*	XXXX	XXXX	XXXX	ELIMINATED	Fornasari and Lora Aprile, 2018; Franceschi et al., 2013.

indicated for moderate pain when NSAIDs are contraindicated.					The item regarding the fixed-dose combination of paracetamol and codeine was eliminated after the first Delphi round. Panellists noted that, although this formulation may provide effective analgesia, its benefit over paracetamol alone is limited, and the risk of opioid-related adverse effects outweighs its routine use at discharge. The discussion emphasised the preference for non-opioid or short-term weak opioid strategies, aligning with a cautious and patient-tailored approach.

SUPPLEMENTARY METHODS

Supplementary Methods. Details of the literature review.

The literature review was conducted on PubMed, ClinicalTrials.gov, Cochrane Database Systematic Reviews to retrieve full-text articles published on humans in the last ten years:

- Metanalysis of randomised controlled trials (RCTs);
- Papers on RCTs;

- Literature reviews;
- Scientific guidelines.

The literature search strategy followed a PICO framework, defining the target Population, Intervention, Comparison and Outcomes to ensure a structured evidence identification process.

The following search strings were used:

- emergency department AND triage AND pain management;
- emergency department AND multimodal analgesia AND pain management;
- analgesia AND treatment AND drugs AND risk AND pain relief;
- analgesia AND (paracetamol OR acetaminophen) AND pain relief;
- analgesia AND (non-steroidal anti-inflammatory drugs OR NSAIDs OR ibuprofen) AND pain relief;
- emergency department AND multimodal analgesia AND (fixed dose combination OR FDC) AND [(paracetamol AND ibuprofen) OR (paracetamol/ibuprofen) OR paracetamol + ibuprofen)] AND pain relief;
- (children OR pediatric OR paediatric) AND emergency department AND pain management AND analgesia; (elderly OR elders) AND emergency department AND pain management AND analgesia.
